# Clinical, Electrocardiographic and Echocardiographic Predictors of Atrial Fibrillation Recurrence After Pulmonary Vein Isolation

**DOI:** 10.3390/jcm14030809

**Published:** 2025-01-26

**Authors:** Aikaterini-Eleftheria Karanikola, Melpomeni Tzortzi, Athanasios Kordalis, Ioannis Doundoulakis, Christos-Konstantinos Antoniou, Ageliki Laina, Panagiotis Tsioufis, Nikos Argyriou, Athanasios Sakalidis, Konstantinos Pamporis, Konstantinos Tsioufis, Dimitrios Tsiachris

**Affiliations:** 1 First Department of Cardiology, Hippokration Hospital, Athens Medical School, National and Kapodistrian University of Athens, Vas. Sofias 114, 11527 Athens, Greece; elinakaranikola@gmail.com (A.-E.K.); akordalis@gmail.com (A.K.); doudougiannis@gmail.com (I.D.); ckantoniou@hotmail.gr (C.-K.A.); agelikilaina@hotmail.com (A.L.); ptsioufis@gmail.com (P.T.); nikos_ar@hotmail.com (N.A.); asakalidis@gmail.com (A.S.); konstantinospab@gmail.com (K.P.); ktsioufis@gmail.com (K.T.); 2 Department of Hygiene, Social-Preventive Medicine & Medical Statistics, School of Medicine, Faculty of Health Sciences, Aristotle University of Thessaloniki, 54124 Thessaloniki, Greece

**Keywords:** atrial fibrillation, catheter ablation, recurrence, predictors, clinical, electrocardiogram, transthoracic echocardiogram

## Abstract

Atrial fibrillation (AF) is a supraventricular arrhythmia and the most common heart rhythm disorder in the adult population worldwide with an estimated prevalence of 2% to 4% of the population. Cases of AF have shown an increasing trend in recent decades, while its frequency is expected to rise even more. Given the significant impact on patients’ quality of life, as well as its major complications, including thromboembolic events, effective rhythm control strategies other than antiarrhythmic medication have emerged, with catheter ablation (CA) being the cornerstone of these. In recent years, CA has been upgraded to a first-line treatment for selected patients. However, complications do exist and arrhythmia-free survival is not always guaranteed. The need to better identify patients more suitable for this specific therapeutic measure is crucial in improving outcomes and preventing arrhythmia recurrences. This review aims to present currently identified predictors of AF recurrence after catheter ablation based on clinical characteristics and electrocardiographic and echocardiographic parameters, in an era of increasing interventional rhythm control approaches for the management of atrial fibrillation.

## 1. Introduction

Atrial fibrillation (AF) is the most prevalent cardiac arrhythmia globally, with a rising incidence due to its progressive nature and increased morbidity and mortality [1]. Atrial remodeling, including dilation and fibrosis, is characteristic of AF and can contribute to structural and electrical changes in the heart. As a result, not only is the burden of AF increased but the risk of recurrence is also elevated, approaching 90% after the initial episode. To improve patient outcomes, the need for effective rhythm control strategies has arisen, and among the available interventions, catheter ablation has emerged [2].

Initially considered a last-resort treatment, catheter ablation (CA) has evolved into a first-line option for selected patients, since it is demonstrating significant benefits in reducing the progression from paroxysmal AF (PAF) to persistent AF and improving quality of life compared to antiarrhythmic drugs [3]. The role of pulmonary vein isolation (PVI) and the reduction in procedural risks led to this transition and thus today, early intervention holds promising results in achieving durable rhythm control and potentially curing AF in individuals [1,4]. The techniques for PVI include traditional thermal methods, such as radiofrequency ablation (RFA) and cryoballoon ablation (CBA), as well as novel non-thermal approaches, such as pulsed-field ablation (PFA) [5].

Despite this progress, the long-term success of catheter ablation remains suboptimal, with recurrence rates ranging from 20 to 50%. Patient variability plays a core role in the outcomes, with underlying atrial myopathy arising as the key contributor to AF recurrence. Atrial fibrosis stabilizes reentrant circuits that sustain AF and therefore relates to persistent arrhythmia and resistance to therapy [6]. In addition, cardiac structural changes such as atrial dilation, inflammation and myocyte injury limit the likelihood of a maintained sinus rhythm. These indicate the importance of timely intervention and the optimization of the procedures [7].

Identifying the predictors of recurrence is crucial for patient selection and treatment strategies. Advances in biomarker research may further the ability to predict recurrence and guide personalized treatment plans, yet it is known that older age, structural heart disease, longer AF duration and larger left atrial size, along with procedural factors such as incomplete PVI, influence patient outcomes [8]. This review article will focus on clinical characteristics, as well as electrocardiographically and echocardiographically derived parameters, associated with AF relapse after CA.

## 2. AF Recurrence After Catheter Ablation

It is important to distinguish AF recurrence from other atrial tachyarrhythmias. A 30 s threshold for AF duration after ablation has been proposed, but this binary classification does not always clearly reflect the clinical outcomes of ablation and the improvement in the patients’ quality of life [4]. AF is considered clinical when it is symptomatic or asymptomatic and recorded on a surface ECG or other recorder and subclinical when it is detected by cardiac implantable electronic devices or smart watches. Recently, the AF burden post catheter ablation has been used as an endpoint by several investigators and may carry a stronger clinical relevance. Up to 50% of patients may experience a recurrence, depending on the method of AF detection, while among these events, 30% may occur very late, after the first year [9]. A large proportion of these patients require a repeat procedure, according to a recently published EHRA survey [10]. Considering the energy used to ablate, the FIRE and ICE and ADVENT randomized trials have proven the non-inferiority of CBA compared to RFA and the non-inferiority of PFA compared to thermal methods, respectively, in the three-month period after CA, regarding recurrent arrhythmias [11,12]. The results from a propensity score-matched comparison are equivalent for arrhythmia-free survival within 1 year, reaching 79.3% with PFA, 74.7% with CBA and 72.4% with RFA [13]. The recently published one-year results from the ADVENT trial, however, may favor PFA, in regard to reduced arrhythmia burden compared to CBA and RFA [14].

**Figure 1:**
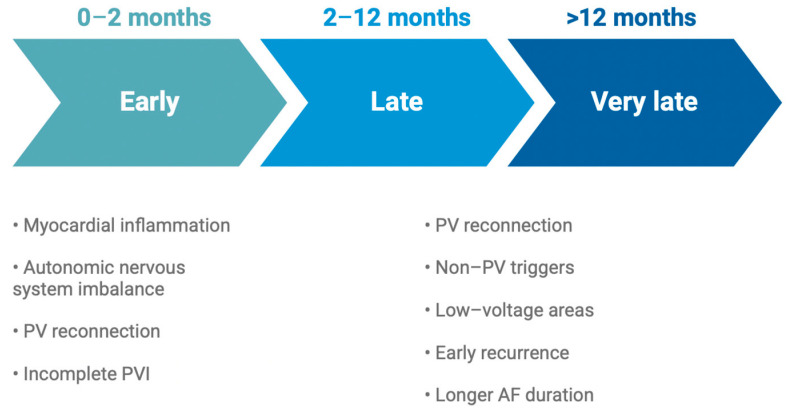
Timing and mechanisms of atrial fibrillation recurrence after catheter ablation. Early recurrence timing was adjusted to fit the recent shortening of the blanking period from three to two months. PV = pulmonary vein, PVI = pulmonary vein isolation, AF = atrial fibrillation.

Based on the timing of AF reappearance post catheter ablation, recurrences are categorized into early, late and very late. Traditionally, early recurrences of AF (ERAFs) were timed within the blanking period, which up until recently was considered as the first three months after ablation, during which time a repeat procedure is advised against [15]. With the recently published 2024 EHRA expert consensus statement on catheter and surgical ablation for atrial fibrillation, the blanking period was reduced to 8 weeks [4]. The mechanisms behind ERAFs are not clearly understood, although myocardial inflammation and injury, as well as a transient imbalance in the autonomic nervous system, seem to play a major part in this phenomenon when it occurs early within the blanking period, while PV reconnection might be of significance when an ERAF occurs later on [16,17,18]. Recurrences outside the first trimester timeframe and up until the first year post ablation are late recurrences (LRAFs). Many investigators have proven a strongly positive relationship of ERAFs with ablation failure, as patients have increased rates of AF after experiencing atrial arrhythmias within the blanking period both with RFA and CBA [19,20]. The number and exact timing of episodes during the first three months also seem to play an important role in the outcome of the procedure [21]. More specifically, ERAFs seem to carry a worse prognosis when occurring further away from the procedure date, owing perhaps to the arrhythmogenic effect of local inflammation and scarring, happening during the first 3–4 weeks [22]. Based on this principle, the shortened duration of the blanking period is expected to reclassify up to 3.4% of procedures as failed [23]. On the other hand, very late recurrences (VLRAFs) after the first twelve months following the procedure are most commonly attributed to PV reconnection and extrapulmonary triggers of AF [24]. Figure 1 shows the main mechanisms of ERAFs, LRAFs and VLRAFs.

## 3. Predictors of AF Recurrence After Catheter Ablation

### 3.1. Clinical Predictors

One of the most important predictors in the long-term success of CA and sinus rhythm maintenance is the AF type and duration. Studies have shown that patients with non-paroxysmal AF (persistent and long-standing persistent) have an increased incidence of post-ablation atrial arrhythmias [25]. In particular, long-standing persistent AF seems to attain the highest rates of recurrence after ablation [26]. An increasing number of researchers are currently advocating for early ablation in hopes of halting AF progression and atrial myopathy. This approach may be associated with the advantage of lower recurrence rates, as well as stroke and congestive heart failure [27]. The benefit is more pronounced if the diagnosis-to-ablation time remains shorter than three years [28]. This observation has prompted cardiovascular societies across both sides of the Atlantic to publish guidelines within the last two years, with an upgraded class I indication of CA as a first-line therapy in selected patients with paroxysmal AF (PAF) [1,4,29].

The recent ESC/EACTS guidelines for the management of atrial fibrillation also emphasize the pivotal role of comorbidities in AF initiation and perpetuation, with the AF-CARE algorithm. More specifically, conditions such as hypertension, heart failure, diabetes mellitus, increased BMI, obstructive sleep apnea and excessive alcohol intake are considered triggers of arrhythmia presentation and should be appropriately managed, in order to prevent recurrences [1]. Furthermore, demographic characteristics, including older age, particularly more than 75 years old, have a positive association with AF relapses after CA [30,31]. The role of gender in AF recurrence rates remains controversial, with some studies suggesting females have decreased arrhythmia-free survival rates and others showing neutral results [32,33]. Chronic kidney disease has also long been linked with worse outcomes regarding rhythm control after CA [34]. A large meta-analysis of 23.468 patients who underwent RFA or CBA showed that the recurrence risk was greater in those patients with impaired renal function [35]. In the case of structural heart disease coexistence, a background of coronary artery disease and cardiomyopathy—such as amyloidosis—increases AF recurrence after CA, with the exception of tachycardia-induced cardiomyopathy [36,37,38,39]. Finally, even though heart failure patients seem to have worse rhythm outcomes after CA, reducing the time to ablation may prevent a reasonable number of recurrences in this group and, therefore, improve quality of life [40,41].

The presence of low voltage areas (LVAs) within the atrial myocardium is a critical indicator of the progression of AF and the stage of the disease in terms of atrial myopathy and remodeling. LVAs have been shown in several studies to be strongly associated with worse clinical outcomes after ablation [42]. Factors such as persistent AF, female sex and increased left atrial volume (LAV) have been independently associated with LVAs [43]. Therefore, predictive scores regarding the presence of LVAs may have clinical value in individualizing the approach, selecting different treatment strategies and improving ablation outcomes in appropriate patients.

Several scores have been developed to predict atrial myopathy and, as a result, the recurrence rates after ablation. Most of these scores use the aforementioned clinical predictors for AF recurrence, sometimes in conjunction with imaging parameters. The CHADS2, CHA2DS2-VASc and R2CHADS2 scores, initially developed for thromboembolic risk prediction, were able to be correlated with both clinical AF recurrences and the presence of LVAs [44,45]. Later, scoring systems were created based on the results of ablation in rhythm control. Among the most promising are the APPLE score and the MB-LATER score, which can also predict late recurrences (>12 months). The APPLE score includes several factors such as age over 65 years, persistent AF, GFR < 60 mL/min/1.73 m^2^, left atrial dilation > 43 mm and ejection fraction < 50%. The MB-LATER score has also been shown to be useful for accurately predicting VLRAFs after RFA or CBA [46,47]. The C2HEST and HATCH scores were also significantly associated with the risk of late recurrence after RFA [48]. Other scoring systems exclusively target the electroanatomical substrate, such as the DR-FLASH score, which—in multiple cohorts—was able to successfully predict those patients who required extensive substrate modification [49,50]. Finally, the LAGO score combined advanced imaging of the LA with CTA or MRA with clinical parameters, such as the AF phenotype and presence of structural heart disease. Values ≥ 3 were correlated with more adverse rhythm outcomes after RFA [51].

To date, no ideal prognostic score has been developed to predict adverse rhythm outcomes after AF ablation. In order to increase the risk stratification accuracy, a combination of more than one of the available scoring systems is suggested [52]. In addition, the integration of laboratory markers, such as BNP levels, into existing clinical scores may increase their predictive value [53]. Table 1 and Table 2 provide a representation of some of the most frequently used clinical scores and the associated variables, as well as a categorization based on the type of recurrence they can predict.

### 3.2. Electrocardiographic Predictors

Various electrocardiographic (ECG) measurements of P-wave parameters have been investigated in AF recurrences after AFCA. A few of the most commonly researched indices are the P-wave duration (PWD), P-wave terminal force in V1 (PTFV1), P-wave dispersion, P-wave amplitude (PWA), P-wave axis, P-wave notching and atrial late potentials. These parameters reflect atrial cardiomyopathy and remodeling, which is a recognized substrate of AF recurrences [63]. Table 3 demonstrates the upper limits of these indices that are related to an increase in AF recurrence after CA.

The P-wave duration is an independent predictor of AF recurrence after ablation according to a recent systematic review and meta-analysis published in *Europace* [64]. Traditionally, the PWD is measured in lead II where the P wave is considered to be better visualized. However, the location of the longest P-wave duration varies from individual to individual. Automated or manual measurement is possible [63]. The PWD is considered normal when measured below 120 ms, while a PWD > 120 ms indicates a partial interatrial block (IAB) in which there is delayed activation of the LA via the Bachmann bundle. In an advanced IAB, there is retrograde activation of the LA via muscle bundles near the atrioventricular junction, and as a result, the P-wave morphology in the inferior leads (II, III, avF) tends to be biphasic with a late negative branch reflecting retrograde conduction in the LA [65]. The risk of AF recurrence increases exponentially with progressively greater PWD. Partial IAB leads to a doubling of recurrences, while advanced IAB leads to a quadrupling. A strong association between the PWD and AF recurrences after ablation was also demonstrated in a 2019 meta-analysis, showing that a PWD > 120 ms to >150 ms in sinus rhythm before PVI leads to recurrence regardless of age, sex, LA dimension and the presence of structural disease [66]. Therefore, the PWD could be a quite useful and easily accessible indicator of underlying atrial remodeling and have clinical significance in better stratifying patients with atrial fibrillation into different treatments [67].

PTFV1 was first described by Morris et al. in 1964 and was initially considered to be an indirect electrocardiographic predictor of left atrial enlargement (LAE) [68]. Since then, several studies have been able to demonstrate a strong association of this marker with interatrial conduction delays, low voltage areas and diffuse atrial fibrosis [69,70]. PTFV1 is defined as the negative deflection at the end of the P wave in lead V1 due to the posterior displacement of the LA. It is measured by multiplying the duration of the terminal negative force of the P wave in lead V1 in seconds by its width in mm. A PTFV1 > 0.04 mm*s is considered pathological. According to a recent meta-analysis, the presence of an abnormal PTFV1 derived from a resting ECG analysis is strongly associated with an increased risk of AF recurrence of at least 23% [71]. A cohort study of 453 patients with persistent atrial fibrillation who underwent ablation for the first time was able to demonstrate a strong relationship between PTFV1 measured 3 months later and AF recurrence [72]. Another prospective study also showed that an abnormal PTFV1 was more frequent in the recurrence group post AFCA, despite normal LA diameter and volume [73]. Finally, increasing values of PTFV1 by more than 0.69 might be able to predict PV reconnection in patients undergoing a second ablation procedure [74].

P-wave dispersion is defined as the absolute difference between the longest and shortest P-wave durations on a 12-lead surface ECG [75]. Although no threshold has been established at which it is considered pathological, a value greater than 40 ms is considered increased, although it can also occur in healthy individuals [76]. The hypothesis that an increased P-wave dispersion could predict paroxysmal AF or recurrence after ablation is based on the assumption that any difference in the P-wave duration in different leads reflects local delays in atrial conduction that could be the manifestation of fibrosis acting as a substrate for AF [77]. A retrospective ECG cohort of 42,751 patients showed that a P-wave dispersion >80 ms had a hazard ratio of 2 for atrial fibrillation when adjusted for age and sex [78]. The results from a meta-analysis of 1674 AF patients showed a significantly increased P-wave dispersion in patients with recurrence after CBA or RFA, suggesting the possible predictive value of this parameter [79].

Changes in the P-wave amplitude (PWA) have been studied post AF catheter ablation, with most researchers agreeing that the PWA decreases, although its correlation with recurrences is still not clear [80]. However, another parameter, the PWD/PWA ratio, has been associated with AF recurrence and low voltage areas (LVAs) in the atrial myocardium, with some researchers suggesting that a ratio of >830 ms/mV has a 61.8% sensitivity and 88.4% specificity for the prediction of AF recurrence [81]. In another study, researchers used a mapping system to identify LVAs and simultaneously measured the maximum PWD to the maximum P-wave height in lead I. This model was statistically significantly associated with recurrences of atrial tachyarrhythmia after ablation [82].

Other P-wave parameters being considered as markers of AF recurrences post catheter ablation are the P-wave axis and notching. While the P-wave axis is one of the most studied parameters for predicting AF risk in the general population—as a reflection of adverse anatomical and electrical atrial remodeling—its predictive value in AF recurrence has not been clearly specified, with studies showing mixed results. Values between 0 and 75° are considered normal [78,83,84,85]. Based on a Japanese study of 249 patients undergoing AF ablation, an abnormal P-wave axis value was identified in 14% of patients and was considered to be independently associated with AF recurrence [86]. On the other hand, the morphological change in the P wave observed in lead II on a 12-lead ECG as M-shaped with a peak-to-peak distance of more than 20 ms is recorded as a notch and reflects the conduction delay caused by LA remodeling and has been shown to predict recurrences in patients who have undergone ablation [87,88].

**Table 3:** ECG P-wave parameters and values associated with atrial fibrillation recurrence after catheter ablation.

P-Wave Parameter	Abnormal Values
P-wave duration (PWD) [65]	>120 ms
PTFV1 [71]	>0.04 mm·s
P-wave dispersion [76,78]	>40 ms or >80 ms
PWD/PWA [81]	>830 ms/mV
P-wave axis [78]	<0 or >75°
P-wave notch [87,88]	Peak-to-peak distance in lead II of more than 20 ms

Finally, a special mention should be made regarding atrial late potentials. These represent low-amplitude, high-frequency electrical signals recorded at the end of the P wave using high-resolution techniques and signal-averaging ECG. They are attributed to delayed and disorganized activity in small areas of the atrial myocardium. The average P-wave signal has been used to predict the risk of developing atrial tachyarrhythmias in patients with structural heart disease [89]. Even though no clear normal limits have been established for the atrium, and it is a rarely studied parameter, owing to the necessity of special equipment, dynamic changes in late atrial potentials have been described after AF ablation [90]. The most commonly used measurements are the duration of the filtered P wave and the root mean square of the potentials in the last 20 ms of the P wave (RMS_20_). The role of this marker in AF recurrences after rhythm control strategies is highlighted by a study of patients undergoing electrical cardioversion [91]. Also, another small study of 15 patients demonstrated that the duration of the P wave as measured by signal-averaging techniques was longer in patients who relapsed after ablation [92].

Considering other ECG features, the presence of a bundle branch block seems to influence patient outcomes. More specifically, both left (LBBB) and right (RBBB) bundle branch blocks have been associated with AF recurrences after ablation [93,94]. Also, the larger amplitude of fibrillatory waves in persistent AF has been shown to be related to better prognosis in a study of 704 patients, given that “fine AF” almost always implies more advanced atrial myopathy [95]. Early repolarization changes, especially in inferior or lateral leads, may also predispose patients to AF recurrence, although the mechanism can only be hypothesized, with possible autonomic dysregulation, ion-channel imbalances and genetic background being held accountable [96]. Particularly in the context of hypertrophic cardiomyopathy, QRS fragmentation and QTc prolongation have been independently associated with arrhythmia recurrences [97]. However, all of these abnormalities need validation in larger cohorts.

### 3.3. Echocardiographic Predictors

The echocardiographic parameters associated with adverse LA remodeling include LA anatomical and functional features, LA synchrony, measures of diastolic function and the presence of atrioventricular valvular heart disease, as well as novel markers, such as the left atrioventricular coupling index, LA stiffness index and right ventricular-to-pulmonary artery coupling (RV-to-PA coupling). Table 4 indicates the respective cut-off values that have been associated with an increase in AF recurrence after CA.

The hallmark of LA anatomical remodeling is LA dilatation, with progressive alteration in mechanical and electrical function and fibrosis [98]. Measures include the anteroposterior LA diameter (LAD) in the parasternal long axis view or the LA volume (LAV) and LA volume index (LAVi) in the apical four- and two-chamber view on a transthoracic echocardiogram (TTE). An increased LAD is closely associated with atrial myopathy and atrial remodeling; therefore, it is considered an independent predictor of AF recurrence after ablation [99,100]. Additionally, its integration in many of the clinical scores previously mentioned has led to their improved sensitivity in predicting recurrences [101]. According to the results of a meta-analysis that examined data from 22 studies and a total of 3750 patients, an increase in the LAD was associated with AF recurrence after ablation [102]. Another recent meta-analysis of 20 studies showed that patients with a larger LAV and LAVi show an increased rate of AF recurrence after RFA ablation [103]. An increased LAV is associated with progressive diastolic dysfunction and consequently reflects a deterioration in LA function leading to a higher risk of AF recurrence after RFA [104]. Finally, the left atrial sphericity index is an important parameter of left atrial geometric remodeling, calculated as the ratio of the maximum transverse to the maximum longitudinal diameter in the apical four-chamber view, and has been associated with AF recurrences after ablation [105,106].

**Figure 2:**
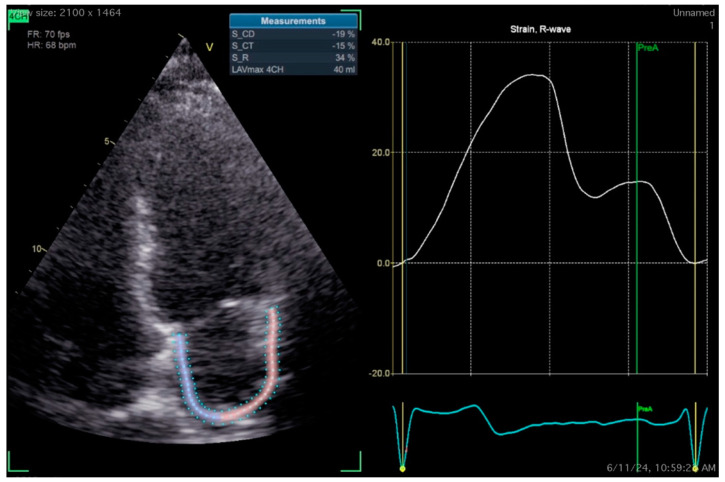
Abnormal left atrial strain parameters by 2D-STE in a 44-year-old male patient with paroxysmal atrial fibrillation in sinus rhythm. Transthoracic echocardiogram was performed 24 h before pulmonary vein isolation with pulsed-field ablation.

Left atrial function is responsible for adequate left ventricular filling. Its assessment by LA strain may be more sensitive than the conventional measurement of LV filling pressures [107,108]. According to a 2018 consensus document of the European Association for Cardiovascular Imaging (EACVI), LA strain—as calculated by 2D speckle-tracking echocardiography (2D-STE)—is a cyclical process that can be subdivided into three phases, the reservoir (LASr), conduit (LAScd) and contraction (LASct), with the latter two phases being valid only for patients in sinus rhythm [109]. Recent studies and meta-analyses have highlighted LA deformation as a potential marker for detecting the population at risk of AF recurrence after ablation [110,111]. Specifically, the CASA-AF study evaluated the three phases of LA deformation in 83 patients with long-term persistent AF before and after ablation. The results showed an improvement in LA function after ablation, with an increase in the LASr and restoration of the LASct, while an impaired LASct after the blanking period appeared to be the only independent predictor of AF recurrence in this population [112]. The recently published ASTRA-AF study examined nine parameters in 132 patients with paroxysmal and persistent AF who underwent PVI with thermal techniques (RFA/cryoablation), including left atrial deformation (LASr, LAScd and LASct). The only parameter with a statistically significant association with AF recurrence was the LASr [113]. Finally, echocardiographically derived LASr has also been corelated with LA-LVAs, thus being a useful risk stratification tool for post-ablation success outcomes [114]. Figure 2 represents a slightly pathological LA strain analysis from a patient with paroxysmal AF in sinus rhythm and the absence of other comorbidities 24 h before CA. Normal ranges have been established by a large meta-analysis of 40 studies and 2542 healthy subjects, although the exact values that hold prognostic implications regarding AF recurrence have not yet been identified [115].

One of the most studied indices of left atrial dyssynchrony is the total atrial conduction time (PA-TDI duration). The PA-TDI duration is a modern echocardiographic parameter for the assessment of the total atrial conduction time and directly reflects both electrical and structural changes in the atria, therefore the extent of atrial remodeling. The measurement is made in sinus rhythm and is the time interval from the onset of the P wave in lead II of the ECG (onset of electrical depolarization) to the maximum A’ wave in tissue Doppler of the lateral wall of the LA (active atrial contraction) [116]. Several studies have shown the correlation of this marker with AF recurrences after ablation [117,118,119,120]. Another indicator of left atrial dyssynchrony is left atrial mechanical dispersion (LA-MD), defined as the standard deviation of the time to maximum positive strain corrected by the R-R interval (SD-TPS, %) [121]. In a study by Sarvari et al., the authors observed that in patients with AF recurrence and normal LA size, the left atrium exhibits significantly greater mechanical dispersion compared with patients after successful CA ablation. Therefore, LA mechanical dispersion may be a useful tool for predicting recurrence in patients with structurally normal hearts [122].

Parameters of diastolic function can be assessed by mitral annular inflow with the use of a pulse wave (PW) and tissue Doppler imaging (TDI) and constitute an indirect measurement of LA pressure. Traditional indicators for assessing left ventricular (LV) diastolic dysfunction include the LV septal (e’_septal_) and lateral (e’_lateral_) wall velocity, the E/e’ ratio, the LA volume index (LAVi) and the maximum tricuspid velocity. Indicators for assessing filling pressures and the degree of diastolic dysfunction are the E/A ratio, the S/D ratio in the pulmonary veins, the DT, the presence of an L wave and the duration of the Ar/A. Based on the above measurements, the left atrial pressure (LAP) can be estimated [123]. Several of the abovementioned markers have been associated with AF recurrence after ablation [124,125,126,127]. In addition, emerging parameters of diastolic dysfunction are currently being considered as predictors of AF recurrence after PVI. One such marker, the left atrioventricular coupling index (LACI), which is the ratio of LA end-diastolic volume to the LV end-diastolic volume, has been associated with AF recurrences post RFA for paroxysmal AF [128]. Finally, the left atrial stiffness index (LASi), as another measure of LA myopathy, is a promising parameter in the prediction of AF recurrence after ablation [129]. Measurement by TTE is possible by dividing the E/e’ ratio by the peak LA strain [(E/e’)/peak atrial longitudinal strain]. Studies have shown that patients with LVAs on voltage mapping had increased LASi, especially when these areas were located in the anterior wall of the left atrium [130].

Other echocardiographic indices being explored in the field include mitral and tricuspid valve regurgitation. Many investigators have investigated the possibility that the presence of functional mitral regurgitation (MR) may lead to increased AF recurrences after PVI. A retrospective study of 132 patients reported that functional MR significantly contributes to the remodeling of the LA substrate, is associated with LVAs and is also statistically significantly associated with AF recurrence after PVI compared with the absence of regurgitation [131]. The results of the EARNEST-PVI study support this hypothesis, as they concluded that in patients with persistent AF and any degree of mitral valve regurgitation, extended PVI methods were superior to simple PVI in preventing recurrences [132]. Tricuspid regurgitation (TR) has also been highlighted as a possible predictor of AF recurrence after RFA, especially when combined with MR [133,134].

Apart from tricuspid regurgitation, other right heart chamber parameters are currently being investigated in AF recurrence post catheter ablation. According to the results of a prospective cohort published in 2023, the AF recurrence rate after ablation in patients with paroxysmal atrial fibrillation is closely related to pulmonary artery pressure (PAP) measured by TTE, although this was not reproduced in the case of persistent AF [135]. This association highlights the importance of pulmonary vascular dysfunction in atrial remodeling and its role in the pathogenesis of AF, as the pulmonary veins are the main source of AF initiation. A novel marker of right ventricular-to-pulmonary artery coupling, as measured by the TAPSE/PAP ratio, was shown to be an independent predictor of the late recurrence of atrial fibrillation after ablation in a group of 203 patients with persistent AF [136]. The size of the right atrium may also influence ablation outcomes, with reports focusing on the right atrial volume (RAV) as a predictor of recurrence [137,138]. A meta-analysis of 12 studies concluded that a higher RAV and RAVi increases the risk of AF after RFA [139]. Finally, right ventricular function is nowadays considered a significant parameter in various heart conditions, including AF outcomes. In a total of 164 patients with paroxysmal, persistent and long-standing persistent AF, an improvement in RV strain values was noted after RFA; however, the absolute change in the RV free-wall strain and four-chamber strain was smaller in patients with AF recurrence, highlighting the importance of RV function in maintaining sinus rhythm [140].

**Table 4:** Echocardiographic parameters and values associated with atrial fibrillation recurrence after catheter ablation.

Echocardiographic Parameter	Recurrence	No Recurrence
LA diameter (LAD) [102]	Variable, >40 mm is enlarged	
LA volume (LAV)/LA volume index (LAVi) [103]	Variable, most agree > 153 mL/>34 mL/m^2^ favors recurrence	
LA sphericity index [106]	>0.678	≤0.678
LA reservoir strain (LASr) [115]	39% (95% CI, 38–41%)	
LA conduit strain (LAScd) * [115]	23% (95% CI, 21–25%)	
LA contraction strain (LASct) * [115]	17% (95% CI, 16–19%)	
Total atrial conduction time (PA-TDI) * [116]	146.7 ± 20.4 ms	130.1 ± 23.0 ms
LA mechanical dispersion (LA-MD) [122]	38 ± 14 ms	30 ± 12 ms
E/A [127]	1.8 ± 0.9	1.5 ± 0.9
DT [127]	214 ± 67 ms	243 ± 68 ms
E/E’ [124]	>14	≤14
L wave [126]	Presence	-
Left atrioventricular coupling index (LACI) [128]	44.0 (43.0–45.0)%	49.5 (47.0–53.0)%
LA stiffness index (LASi) [129,130]	0.83 ± 0.46 or 1.64 ± 1.70	0.40 ± 0.22 or 0.61 ± 0.46
Mitral/tricuspid regurgitation (MR/TR) [134]	More than mild	-
Pulmonary artery pressure (PAP) [135]	≥35 mmHg	<35 mmHg
TAPSE/PAP [136]	≤0.57	>0.57
Right atrial volume (RAV) [138]	≥87 mL	

* indicates parameters measured only during sinus rhythm.

## 4. Discussion

Despite numerous studies in recent years regarding AF ablation strategies, prognostic markers and outcomes, major questions around AF recurrence remain unanswered. For example, the recent shortening of the blanking period from 3 months to 8 weeks may be more reflective of the pathophysiological processes occurring after CA and recognize patients in need of adjunctive therapy earlier, therefore offering improved outcomes. However, there are reports suggesting that when ablating with certain technologies, such as pulsed-field ablation (PFA), an even shorter blanking period of one month may be adequate, as patients presenting with an ERAF in the second or third month after CA have a high chance of undergoing a redo procedure [141]. Furthermore, as electroporation was not widely used for cardiac arrhythmia ablation up until recently, there is a lack of sufficient data regarding AF recurrence predictors after ablation with the specific type of energy and potential differences in comparison to thermal forms. Even though it is too soon to hypothesize about long-term outcomes, lesion durability and VLRAF rates, the initial results seem promising. Preliminary data from the IMPULSE, PEFCAT and PEFCAT II trials and the EUPORIA registry suggest high rates of freedom from AF 12 months after the index procedure, between 74 and 85% [142,143].

The future seems promising in the field of predicting recurrences, particularly concerning the incorporation of artificial intelligence (AI) into pre-ablation planning. Recent research suggests that AI-enabled pre-ablation computed tomography (PVCT) combined with clinical variables can accurately predict AF and demonstrates a notable improvement over traditional predictive methods [144]. Other applications of AI in imaging parameters include the prediction of atrial wall stretch and its relationship with AF recurrence after CA [145]. Regarding electrocardiographic parameters, AI algorithms that can effectively analyze ECG data prior to ablation and predict the risk of recurrence have also been described, therefore providing physicians with the ability to develop patient-specific interventions [146]. Machine learning models have proved to be effective or even superior compared to conventional statistical methods in predicting AF recurrence post ablation, owing to their ability to deploy extensive data to reveal complex patterns and associations, offering a deeper understanding of factors related to AF recurrence [147,148]. However, challenges remain, as the inclusivity of all different patient populations when training machine learning models is sometimes questioned, along with ethical and data safety considerations regarding AI use in clinical practice [149].

Given the novelties in electrocardiographic and echocardiographic parameters, along with the accessibility and widespread use of these diagnostic tools across all kinds of socioeconomic backgrounds, the current clinical scores for predicting AF recurrence after CA almost seem outdated. The incorporation of these parameters with basic clinical characteristics into a single predictive model sounds exceptionally promising and could aid in achieving a more personalized therapeutic strategy for all AF patients, depending on their risk for recurrence after CA, thereby improving the long-term success of the procedure. In addition, abnormal P-wave values may reflect a greater extent of atrial myopathy and have frequently been associated with an increased risk of stroke [83,150,151]. Furthermore, reduced LA strain values have been associated with the progression of AF from paroxysmal to persistent, an increased risk of left atrial appendage thrombus formation, increased left ventricular filling pressures and diastolic dysfunction [152,153,154,155]. These patients may be candidates for aggressive risk factor and comorbidity management and a lower threshold for anticoagulation discontinuation. Particularly in cases of moderate thromboembolic risk, where there is lack of robust data regarding tailored anticoagulation therapy after catheter ablation, these easily acquired markers may be of prognostic significance. However, limitations do exist. To date, these markers have not been validated in large cohorts, and normal values have not been clearly established. In addition, the manual measurement of P-wave parameters may be technically challenging, time-consuming and subject to interobserver variability [63]. Finally, left atrial strain requires high-quality 2D imaging, which is not always possible for all patients. Furthermore, reproducibility issues have arisen, especially considering the use of different vendors and software for calculation [156].

## 5. Conclusions

Atrial fibrillation recurrence following catheter ablation remains a significant challenge, with clinical, ECG and echocardiographic parameters playing pivotal roles in its prediction. Early recurrences often result from transient procedural effects, whereas late and very late recurrences are more indicative of structural remodeling and pulmonary vein reconnection. While no single predictor is sufficient, the integration of clinical scores, electrocardiographic markers, such as P-wave parameters, and left atrial echocardiographic indices provides promising tools for enhancing prediction accuracy and guiding treatment strategies. Looking ahead, there is a lot of progress to be made in the field. The incorporation of AI is being introduced, and a new area of exploration emerges. By advancing predictive tools and embracing innovative technologies, the field is well positioned to meet these challenges and improve patient treatment strategies and, therefore, outcomes.

## Figures and Tables

**Table 1:** Commonly used clinical scores to predict atrial fibrillation recurrence after catheter ablation.

Score	Parameters Included						
CHADS_2_	CHF	Age ≥ 75	DM	TIA/stroke			
CHA_2_DS_2_-VASc [45]	CHF	Age ≥ 75	DM	TIA/stroke	Vascular disease	Age ≥ 65	Female
R_2_CHADS_2_ [45]	CHF	Age ≥ 75	DM	TIA/stroke	Renal dysfunction		
APPLE [54]	Age > 65	Persistent AF	eGFR < 60	LAD ≥ 43mm	LVEF < 50%		
MB-LATER [55]	Male	BBB on ECG	LAD > 47mm	ERAF	Persistent AF		
C2HEST [48]	CAD/COPD	HTN	Age > 75	HF	Thyroid disease		
HATCH [48]	HTN	Age ≥ 75	TIA/stroke	COPD	HF		
DR-FLASH [49]	DM	CKD	Persistent AF	LAD > 45mm	Age > 65	Female	HTN
PLAAF [56]	Persistent AF	LA area	Abnormal PV anatomy	AF history	Female		
BASE-AF_2_ [57]	BMI > 28	LAD > 40mm	Smoking	ERAF	AF duration > 6 years	Non-PAF	
ATLAS [58]	Age > 60	Female	Non-PAF	Smoking	LAVi		
CAAP-AF [59,60]	CAD	LAD > 40	Age > 50	Persistent AF	AAD failure	Female	
SCALE-CryoAF [61]	SHD	CAD	LAD > 43mm	LBBB	ERAF	Non-PAF	
LAGO [51]	SHD	AF type	CHA2DS2-VASc ≤ 1	LAD > 40mm	LA sphericity		
ACEF [62]	Age	Creatinine	LVEF				

CHF = congestive heart failure, DM = diabetes mellitus, TIA = transient ischemic attack, LAD = left atrial diameter, LVEF = left ventricular ejection fraction, BBB = bundle branch block, ERAF = early recurrence of AF, CAD = coronary artery disease, COPD = chronic obstructive pulmonary disease, HTN = hypertension, CKD = chronic kidney disease, PV = pulmonary vein, PAF = paroxysmal AF, AAD = antiarrhythmic drug, SHD = structural heart disease, LBBB = left bundle branch block.

**Table 2:** Commonly used clinical scores and type of recurrence after catheter ablation.

Score	Ablation Strategy	Type of Recurrence
CHADS_2_	RFA	ERAF, LRAF
CHA_2_DS_2_-VASc	RFA	ERAF, LRAF
R_2_CHADS_2_	RFA	ERAF, LRAF
APPLE	RFA	LRAF, VLRAF
MB-LATER	RFA, CBA	LRAF, VLRAF
C2HEST	RFA	LRAF
HATCH	RFA	LRAF
DR-FLASH	RFA	Substrate
PLAAF	CBA	LRAF, VLRAF
BASE-AF_2_	CBA	LRAF, VLRAF
ATLAS	RFA	Any
CAAP-AF	RFA, CBA	LRAF
SCALE-CryoAF	CBA	VLRAF
LAGO	RFA	Any
ACEF	RFA	LRAF

RFA = radiofrequency ablation, CBA = cryoballoon ablation, ERAF = early recurrence of AF, LRAF = late recurrence of AF, VLRAF = very late recurrence of AF.
